# Prevalence and Risk Factors for Antimicrobial Resistance of *Mycoplasma genitalium* Infections in a High-Risk Population

**DOI:** 10.3390/jcm13164924

**Published:** 2024-08-21

**Authors:** Asher Hackett, Orit Yossepowitch, Yael Goor, Rivka Sheffer, Orna Schwartz, Yonatan Sheftel, Yarden Weiss, Yasmin Maor

**Affiliations:** 1 Division of Dermatology, Rabin Medical Center, Petah-Tikva 4941492, Israel; 2 Infectious Disease Unit, Edith Wolfson Medical Center, Halochamim 62, Holon 5822012, Israel; orityoss@netvision.net.il; 3 Levinski Clinic of the Tel Aviv District Office, Ministry of Health, Tel Aviv 6699001, Israel; yael.goor@moh.gov.il; 4 Tel Aviv Health District, Ministry of Health, Tel Aviv 6473904, Israel; rivka.sheffer@telaviv.health.gov.il; 5 Microbiology and Immunology Laboratory, Edith Wolfson Medical Center, Holon 5822012, Israel; ornas@wmc.gov.il; 6 Department of Pediatrics, Edith Wolfson Medical Center, Holon 5822012, Israel; yonatansh3@clalit.org.il; 7 Faculty of Medical and Health Sciences, Tel Aviv University, Tel Aviv 6997801, Israel

**Keywords:** *Mycoplasma genitalium*, sexually transmitted infections, men who have sex with men, resistance, antibiotics

## Abstract

**Background/Objectives:** *Mycoplasma genitalium* (*MG*) infections and antibiotic resistance are increasing in prevalence while treatment options are limited. Limited data exist regarding *MG* resistance in Israel. Our aim was to study the prevalence of *MG* resistance in a sexually transmitted infection (STI) clinic in Israel. **Methods:** We performed a single-center retrospective study among patients attending an STI clinic during 2019–2020. *MG* isolates were tested to detect their resistance to azithromycin and fluoroquinolones (FQs) using commercial kits (Allplex™ *MG* & AziR Assay, Allplex™ *MG* & MoxiR Assay). We collected patient data regarding the risk factors for STIs and resistance. A multivariate logistic regression model was used to identify the risk factors for resistance. **Results:** Of the 142 patients who tested positive for *MG*, 50 (35.2%) and 22 (15.5%) had resistant mutations to azithromycin and FQ, respectively, and 13 (9.2%) showed resistance to both agents. In a multivariate logistic regression model, men who have sex with men (RR 7.01 95% CI 3.00–16.33) and past STIs (RR 2.33 95% CI 1.01–5.34) were independent risk factors for azithromycin resistance. **Conclusions:** We found a high prevalence of azithromycin resistance and, to a lesser degree, FQ resistance. These findings may help design the treatment guidelines and support routine resistance testing in high-risk populations.

## 1. Introduction

*Mycoplasma genitalium* (*MG*) is an emerging pathogen in sexually transmitted infections (STIs). The most common presentation in men is urethritis, and it is associated with cervicitis and pelvic inflammatory disease (PID) in women [1]. Some reports have suggested an association between *MG* and preterm birth [2]. It may also present as an asymptomatic infection in all sexes. The prevalence of *MG* varies geographically and according to risk factors. In a national survey aiming to represent the general population in the US, the prevalence of *MG* was 1.8% for men and 1.7% for women in the years 2017–2018 [3]. In a prospective study of Israeli men (*n* = 259) tested at our sexual health facility between 2008 and 2010, *MG* had an overall prevalence of 6.6% and a prevalence of 11.9% in patients with symptoms [4]. An increase in the prevalence of *MG* over the years has been reported. In a large retrospective Danish study (*n* = 31,600), the detection rate of *MG* in nuclear acid amplification testing (NAAT) increased significantly over the years 2006–2010 from 7.9% to 10.3% for men, and from 2.4% to 3.8% for women [5]. Some of the reported increase in *MG* prevalence may be related to the spread of NAAT testing and advocating screening for *MG* in asymptomatic people with risk factors for STIs [5]. 

*MG* is a mollicute bacteria, i.e., it lacks a cell wall, meaning it is innately immune to cell-wall-targeting antibiotics, such as beta-lactams and vancomycin [6]. Only a few antibiotic classes have activity against *MG*. These include macrolides, fluoroquinolones, and tetracyclines. Azithromycin has an 85–95% cure rate in susceptible strains. However, substantial macrolide resistance is being reported worldwide due to mutations in the 23S rRNA gene [7,8,9]. In a large USA study, resistance to macrolides was detected in up to 50% of samples [10]. Data are emerging regarding resistance to the once-considered highly effective, second-line treatment with moxifloxacin, which is an agent of the fluoroquinolone (FQ) class. In an Australian study, 15% of samples had a mutation in the parC or gyrA genes associated with FQ resistance [7]. These mutations have been linked with microbiological treatment failure and persistent symptoms [11]. 

It has been postulated that the well-established empirical treatment of non-gonococcal urethritis with single doses of ceftriaxone and azithromycin may be selected for resistance to azithromycin in *MG* [12,13]. Due to widespread macrolide resistance, 2021 European guidelines now recommend macrolide resistance testing on all positive *MG* samples. Routine fluoroquinolone resistance testing is not recommended, though may be useful in patients with documented moxifloxacin treatment failure [12]. These guidelines recommend extended macrolide treatment in wild type *MG* or when resistance testing is unavailable. Fluoroquinolones are recommended in macrolide-resistant cases [12]. Studies from a Melbourne clinic have reported a high rate of microbial cure with doxycycline as the first agent to reduce microbial load, followed by a resistance test guided by azithromycin or moxifloxacin [14,15]. Based on these and other studies, CDC 2021 guidelines currently recommend resistance-guided therapy: sequential doxycycline therapy followed by azithromycin if sensitive or moxifloxacin if resistant to macrolides or if macrolide testing is unavailable [16]. Although *MG* is prevalent in Israel [17], routine resistance testing for *MG* is not available. Therefore, there is limited information regarding the resistance patterns of *MG* in Israel, particularly in high-risk populations. In this study, we tested for macrolide and FQ resistance in patients at Levinski Clinic, a free-of-charge sexual health center in Tel Aviv, Israel, which offers routine STI NAAT testing. The Levinski Clinic in Tel Aviv caters to the greater vicinity of the Tel Aviv area and is the largest sexual health center in Israel. The majority of STIs in the Tel Aviv area are diagnosed by this clinic.

## 2. Patients and Methods

### 2.1. Sample Population

This study was approved by the Edith Wolfson Medical Center institutional review board (0116-20-WOMC). This retrospective study was performed on samples from patients who approached the Levinsky clinic between the years 2019 and 2020. The Levinsky clinic is a sexual health clinic located in Tel Aviv, the largest city in Israel. All people attending this clinic are tested for STIs. Most people attending this clinic are at high risk for STIs. The basic screening for symptomatic and asymptomatic individuals includes serological testing for human immunodeficiency syndrome (HIV), syphilis, and nucleic acid amplification tests (NAATs) of urine samples for *Neisseria gonorrhoeae*, *Chlamydia trachomatis*, and *MG*. In addition, rectal, throat, vaginal, and urethral specimens are collected for testing according to the patient’s reported sexual practices and symptoms. 

In this study, we included symptomatic and asymptomatic individuals who attended the Levinsky clinic between 1 January 2019 and 31 December 2020, and tested positive for *MG* by means of an NAAT from any site. 

### 2.2. STI Testing

Patients underwent testing for STIs as part of the routine practice of the Levinsky clinic. Urine, rectal, throat, vaginal, and urethral specimens were collected by swabs. Most attendees performed self-swabbing at the clinic, and if that was not possible, a physician or a nurse at the clinic took the sample. The samples were immediately placed in a universal transport medium (Copan, Murrieta, CA, USA) and were transferred at room temperature to the microbiology laboratory at the Edith Wolfson Medical Center. DNA for analyses was extracted using the NUCLISENS easyMAG system (Biomerieux, Boston, MA, USA) according to the manufacturer’s instructions. Thereafter, the DNA was frozen at −80 °C for further analysis. The presence of *C. trachomatis*, *N. gonorrhoeae*, and *MG* was assessed by a multiplex polymerase chain reaction (PCR) (Allplex^TM^ *CT*/*NG*/*MG*/*TV* Assay, Seegene Inc., Seol, Republic of Korea), which conforms to the European diagnostic standards for in vitro diagnostics (CE-IVD), certified for use in Europe and the USA, according to the manufacturer’s instructions. All tests included both a negative control and a positive control for each pathogen. 

### 2.3. Antibiotic Resistance Screening

The analysis of antimicrobial resistance to azithromycin and FQs was performed after collecting all the samples. DNA was thawed, and the presence of resistance to azithromycin and FQ in *MG* isolates was analyzed by PCR using commercial kits. Allplex™ *MG* & AziR Assay (Seegene Inc., Seol, Republic of Korea) was used to detect resistance mutations to azithromycin (A2059T, A2058T, A2058C, A2058G, A2059C, and A2059G) according to the manufacturer’s instructions. Allplex™ *MG* & MoxiR Assay (Seegene, Inc., Seol, Republic of Korea) was used to detect resistance mutations to FQ (A247C, G248A, G248T, G259A, G259C, G259T) according to the manufacturer’s instructions. The PCR reactions were run on the CFX96™ Real-Time PCR Detection System (Bio-Rad Laboratories, Hercules, CA, USA) and analyzed using Seegene Viewer for Real-Time Instruments software V3.23.000 (Seegene Inc., Seol, Republic of Korea).

### 2.4. Statistical Analysis

To describe the population, we used mean and medians for continuous variables and numbers and percentages for dichotomous variables. Variables were compared using Student’s *t*-test, Mann–Whitney’s U test, Pearson’s chi-squared test, ANOVA, and Fisher’s exact test as appropriate. All statistical tests were 2-sided. *p*-value < 0.05 was defined as statistically significant for analyses. Risk factors for resistance were assessed using logistic regression. Independent variables found to be significantly associated with the dependent variable in bivariate analysis were entered into backwards, conditional multivariate logistic regression analysis, with results presented as the odds ratio (OR) with a 95% confidence interval (CI). Data were analyzed using SPSS Statistics for Windows, Version 26.0 (Armonk, NY, USA: IBM Corp., 2020).

## 3. Results

This study included 142 patients who tested positive for *MG*. Patients’ characteristics are detailed in Table 1. Twenty-four patients had two positive samples, and two patients had three positive samples from multiple anatomical locations. Eight patients had samples from multiple dates, and the latter dates were considered to be tests of cure. These samples were removed from the primary analysis, which determined the prevalence of antimicrobial resistance. The mean age was 31.7 years, and 35.2% were females. This study included 38 (26.8%) symptomatic patients. As can be seen in Table 1, 46.5% had a past history of an STI, and 13 patients (9.2%) had received past antibiotic treatment. Men who have sex with men (MSM) comprised 46.5% of this cohort, and 80.3% of subjects had sexual relationships in the past with more than 20 partners. About a fourth (28.8%) had an additional concomitant STI, where *C. trachomatis* was the most common (18.2%).

### Resistance

**Figure 1:**
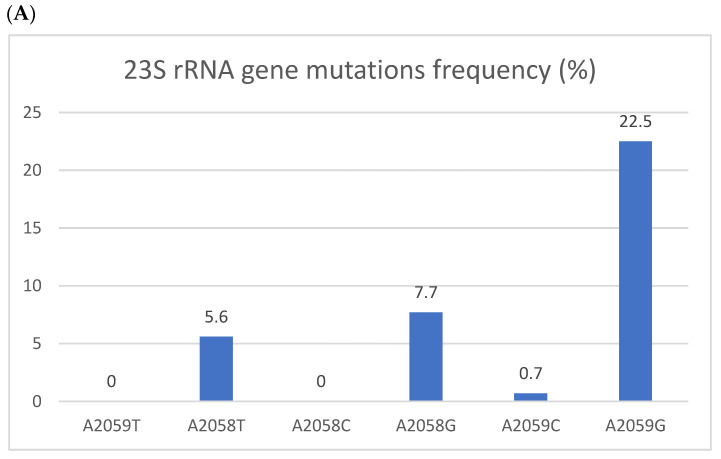

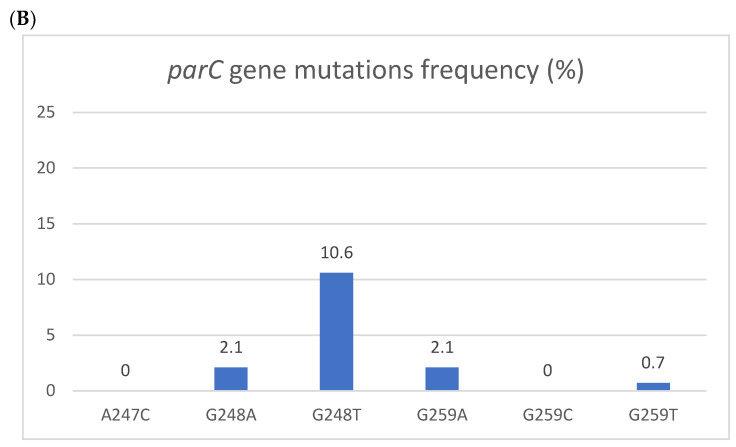
Resistant mutations to azithromycin (**A**) and fluoroquinolones (**B**). Resistant mutations to azithromycin (**A**) reflected by the 223S rRNA gene mutation and fluoroquinolones (**B**) reflected by *parC* gene mutations. The Y axis denotes the percentage of patients, and the numbers above the bars are percentages. Some isolates had more than one mutation.

Antibiotic resistance was common. In total, 50 of the 142 patients (35.2%) had at least one azithromycin-resistant mutation, and 22 (15.5%) had an FQ-resistant mutation. Thirteen patients (9.2%) showed dual resistance mutations to both agents. Mutation frequencies are depicted in Figure 1. In a bivariate analysis (Table 2), male gender, a past STI, MSM, rectal infection, having more than one site involved with *MG,* receiving money for sex, and substance use were associated with azithromycin resistance mutations. Having fewer sexual partners was associated with wild-type *MG*. However, in a multivariate logistic regression model (Table 2), only MSM (RR 7.011 95% CI 3.009–16.336) and a past STI (RR 2.333 95% CI 1.018–5.347) were independent risk factors for azithromycin resistance. In our cohort, 59.4% of MSM and 15.4% of patients who were not MSM had an azithromycin-resistant mutation (*p* < 0.001). Eleven of the thirteen patients with resistance mutations to both azithromycin and FQ were MSM. A similar multivariate analysis on FQ resistance was not conducted due to the low number of resistant samples. 

Test of cure (TOC) data were available for 48 patients, and 10/48 (20.8%) were positive for *MG*. Of the positive patients on TOC, one originally had wild-type *MG*, seven had azithromycin resistance mutations, one had FQ resistance, and one patient had dual resistance. Nine of the ten patients with a positive TOC were MSM. Resistant mutations for azithromycin were detected in 10/38 (26.3%) patients who were negative on TOC, of which five had dual resistance. Two patients had the *parC* mutation only.

## 4. Discussion

In this single-center cohort, we discovered substantial *MG* resistance to azithromycin (35.2%) and, to a lesser degree, to FQ (9.2%). These results are similar to those reported in other countries [11,12,18,19,20]. In a systematic review and meta-analysis, the prevalence of azithromycin resistance was 35.5%. Prevalence increased from 10% before 2010 to an average of 51% in 2016–2017. FQ resistance remained stable over that time at 7.7% [18]. These resistances have been linked to treatment failure [11,19] and highlight the importance of resistance-guided therapy, as is now recommended [12,20].

Azithromycin inhibits protein synthesis by binding to the A2058 and A2059 residues of 23S rRNA or close to the peptidyl transferase site (V region) of the 50S ribosomal subunit. Binding results either in the inhibition of transfer of the transfer RNA (tRNA) from the aminoacyl site to the V region, or the dissociation of tRNA at this site. Resistance occurs by target modification through the single nucleotide polymorphism (SNP) and, therefore, is easily acquired [21,22,23,24,25]. In our cohort, multiple patient characteristics were associated with azithromycin resistance in the bivariate analysis, but only MSM patients were at higher risk for azithromycin resistance in the multivariate analysis. A high prevalence of azithromycin resistance has been reported elsewhere in this population. In a report for a clinic in Sydney, 75% of MSM patients with a positive *MG* test had an azithromycin resistance mutation [26]. In a French study, the overall rate of *MG* resistance (prevalent and incident cases) to azithromycin was 67.6% [27]. 

Quinolones rapidly inhibit bacterial DNA synthesis, an event that is followed by rapid bacterial cell death. They inhibit the enzymatic activities of two members of the topoisomerase class of enzymes—DNA gyrase and topoisomerase IV. Reports have shown that moxifloxacin resistance results from mutations in the quinolone resistance-determining region of either the *parC* gene (topoisomerase IV) or the *gyrA* gene (DNA gyrase) [28,29,30]. The most prevalent *parC* mutation detected in our cohort was G248T, which confers to the amino acid change S83I. This SNP affecting the serine amino acid at position 83 is the most common SNP. In Australia and in other reports, the prevalence of this mutation increased significantly over the years 2012–2020 [19]. This SNP is associated with treatment failure [11], and it has been suggested that its increasing prevalence indicates that the treatment may be selected for this variation [18]. Unfortunately, most of our patients did not return for a TOC, and we do not have enough data to reflect the relationship between resistance development over time and treatment outcome.

There are no commercial tests available that target *gyrA* mutations, and therefore, we did not test for *gyrA* mutations. Our understanding of the molecular pathways for FQ resistance in *MG* is still evolving. Earlier studies showed an uncertain role of *gyrA* in FQ resistance, perhaps due to the small sample size and a relatively low prevalence of these mutations [29,31]. In a recent 2023 large Australian cohort, *gyrA* SNPs were less common than *parC* changes and were associated with treatment failure, which showed a synergistic effect when combined with a corresponding *parC* mutation [11]. If *gyrA* testing becomes more widespread, we may be able to gain a further understanding of the role of these mutations on resistance.

In recent years, in attempts to decrease the rise in STIs in high-risk populations, screening for STIs has become common. It is assumed that screening can be effective in reducing the prevalence of all STIs. A recent review by Kenyon et al. challenges this assumption regarding *N. gonorrhoeae*, *C. trachomatis*, and *MG* [32]. There is little evidence that screening for *MG* indeed reduces the prevalence of this pathogen. In addition, there is growing evidence regarding the harm of screening asymptomatic individuals related to increased antibiotic consumption and increased resistance to *MG*, as well as to *N. gonorrhoeae* and *C. trachomatis.* Increased antibiotic consumption has a deleterious effect on the microbiome. Screening may also negatively affect the well-being of the patients [32]. As a result, some agencies have recommended testing only symptomatic patients [12,33]. Our results highlight the problem of antibiotic resistance in *MG* isolates in high-risk patients and emphasize the complexity regarding whether patients indeed benefit from screening. 

Limitations to our study include selection bias and information bias. The healthcare system in Israel is universal and single-payer, i.e., all citizens have access to free primary care, including sexual health services. The Levinski sexual health clinic is unique in that it provides free and anonymous care to all. It caters to people with health insurance who require anonymous sexual health services, to non-citizens who do not have health insurance, including asylum seekers, as well as to other populations with reduced access to healthcare services, such as people with substance abuse issues and sex workers. This bias was reflected in our results, where, for example, 19% reported receiving money for sex and 45% were MSM. Thus, our findings may not be representative of the entire population of Israel and are more comparable to results reported by other sexual health clinics elsewhere. Information regarding patient history was gathered before testing. Patients were offered anonymity, which aided in collecting personal information that patients might have been hesitant to share otherwise. However, even in an anonymous setting, patients may elect to keep information private and not share certain details with the provider, which may influence subgroup analysis. We were also unable to corroborate the demographic data with an insurance provider, as is often performed in studies with an identified or a de-identified population. 

In summary, in our high-risk cohort, we found a high prevalence of in vitro antibiotic resistance of *MG* mostly to azithromycin and, to a lesser extent to FQ, with MSM being a risk factor for resistance. These findings are alarming due to the limited treatment options available for *MG*. Future directions include assessing trends in resistance through the years and assessing prevalence and resistance in the general population in Israel. These findings may help design screening programs and treatment guidelines in Israel and support the routine use of resistance testing in high-risk populations. 

## Figures and Tables

**Table 1:** Patient characteristics.

Characteristic	N (%)
Number of patients	142
Mean age	31.7 (SD 9.3)
Gender—female	50 (35.2%)
Symptomatic	38 (26.8%)
History of STI	66 (46.5%)
Previous antibiotic treatment	13 (9.2%)
MSM	64 (45.1%)
More than 20 lifetime sexual partners	114 (80.3%)
More than 20 sexual partners in past three months	37 (26.1%)
Reported receiving money for sex	27 (19%)
Reported substance use	58 (40.8%)
HIV positive	7 (4.9%)
Concomitant STI	41 (28.8%)
Concomitant *Chlamydia trachomatis*	26 (18.2%)
Concomitant *Neisseria gonorrhoeae*	15 (10.5%)

**Table 2:** Multivariate logistic regression (*n* = 142) assessing risk factors for azithromycin resistance.

	Bivariate Analysis	Multivariate Analysis	
	*p* Value	RR * (95% CI)	*p* Value
More than one site involved	0.05		
Urinary infection	0.544		
Rectal infection	0.038		
Throat infection	0.534		
Concomitant *Chlamydia trachomatis*	0.889		
Concomitant *Neisseria gonorrhoeae*	0.945		
Male gender	<0.001		
Symptoms	0.584		
Past STI *	<0.001	2.333 (1.018–5.347)	0.45
Previous antibiotic treatment	0.386		
MSM *	<0.001	7.011 (3.009–16.336)	<0.001
20 or more lifetime sexual partners	<0.045		
5 or less sexual partners in the last three months	0.001	0.685 (0.494–0.950)	0.24
Reported receiving money for sex	0.014		
Substance use	0.046		
HIV * status	0.356		

* Abbreviations: RR—relative risk; STI—sexually transmitted infections; MSM—men who have sex with men; and HIV—human immunodeficiency virus.

## Data Availability

Data is available from the authors upon request.
